# Glycosciences.DB: an annotated data collection linking glycomics and proteomics data (2018 update)

**DOI:** 10.1093/nar/gky994

**Published:** 2018-10-24

**Authors:** Michael Böhm, Andreas Bohne-Lang, Martin Frank, Alexander Loss, Miguel A Rojas-Macias, Thomas Lütteke

**Affiliations:** 1 Institute of Veterinary Physiology and Biochemistry, Justus-Liebig University Giessen, Frankfurter Str. 100, 35392 Giessen, Germany; 2 Medical Faculty Mannheim, University Heidelberg, Ludolf-Krehl-Str. 13–17, 68167 Mannheim, Germany; 3 Biognos AB, Generatorsgatan 1, Box 8963, 40274 Göteborg, Sweden; 4 Gebrüder Gerstenberg GmbH & Co. KG, EDV, Rathausstraße 18–20, 31134 Hildesheim, Germany

## Abstract

Glycosciences.DB, the glycan structure database of the Glycosciences.de portal, collects various kinds of data on glycan structures, including carbohydrate moieties from worldwide Protein Data Bank (wwPDB) structures. This way it forms a bridge between glycomics and proteomics resources. A major update of this database combines a redesigned web interface with a series of new functions. These include separate entry pages not only for glycan structures but also for literature references and wwPDB entries, improved substructure search options, a newly available keyword search covering all types of entries in one query, and new types of information that is added to glycan structures. These new features are described in detail in this article, and options how users can provide information to the database are discussed as well. Glycosciences.DB is available at http://www.glycosciences.de/database/ and can be freely accessed.

## INTRODUCTION

Carbohydrates, often referred to as glycans, are one of the four major classes of biomolecules, next to nucleic acids, proteins and lipids. Of these, carbohydrates are the most abundant and also the most complex molecules (1). Besides their well-known functions as energy storage or structural components they are parts of glycoproteins or glycolipids and cover cell surfaces in the glycocalyx (2,3). Here, they serve as recognition sites for cell–cell and cell–matrix interactions but also for pathogens such as viruses, which frequently interact with glycans on the cell surface to enter their host cells. Glycans are also involved in immune responses, inflammation and diseases such as cancer (2–4). Carbohydrates are often specifically recognized. For example, human and avian influenza viruses recognize their hosts by specific glycan motifs (5,6). Therefore, researchers in glycomics-related projects need to be able to find information on the specific glycans they are interested in.

*Glycosciences.DB*, formerly known as *SweetDB* (7), was one of the first efforts to collect information on carbohydrate structures and make them available online. The database is part of the *Glycosciences.de* web portal (8), where it is freely accessible at http://www.glycosciences.de/database/. Initially seeded with data from the discontinued Complex Carbohydrate Structure Database (CCSD, often referred to as *CarbBank*) (9,10), further information has been added over the years, such as 3D structure models generated by *Sweet-II* (11), nuclear magnetic resonance (NMR) spectra imported from *SugaBase* (12) or manually entered from the literature, or links to entries of the worldwide Protein Data Bank (wwPDB) (13) that feature carbohydrates. Currently, the wwPDB is the main source of new data in *Glycosciences.DB*. At the time of writing, *Glycosciences.DB* contains ∼25 000 glycan structure entries with 12 500 3D structure models, 20 000 literature references, 3400 ^1^H or ^13^C NMR spectra and more than 10 000 references to carbohydrate-containing wwPDB entries.

In 2018, a major update of the *Glycosciences.de* portal has been released, which not only puts a more modern design to the portal but also adds a series of new functionality to *Glycosciences.DB*, including improvements in search features and in display of information.

## NEW FEATURES IN GLYCOSCIENCES.DB

### Entry handling

**Figure 1.**
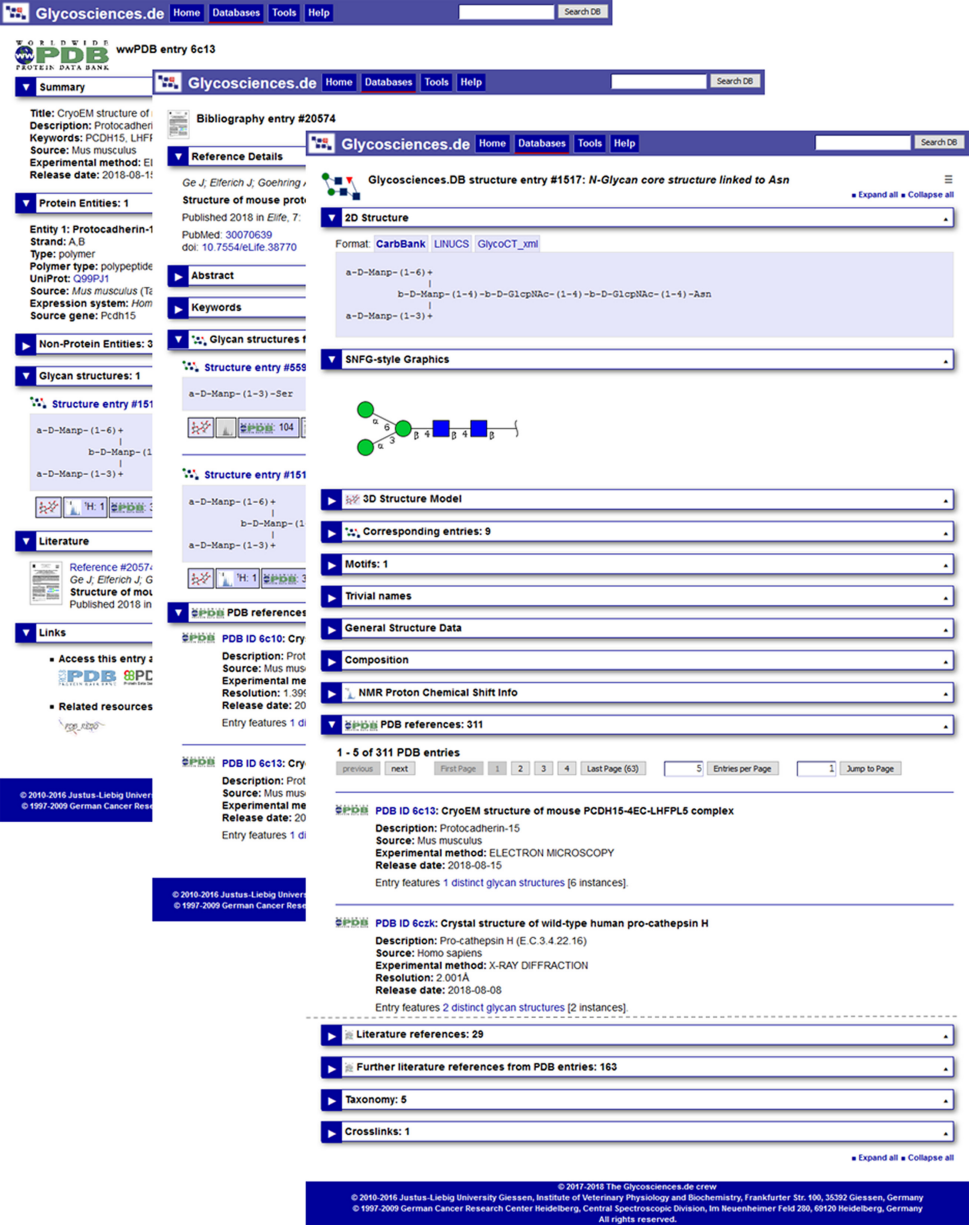
Screenshots of *Glycosciences.DB* glycan structure entry (front, truncated at the dashed line), literature entry (middle) and wwPDB entry (back). All three entries are linked to each other: The wwPDB entry contains both the displayed N-glycan core structure entry and the literature reference. No glycan structure is registered yet with the literature entry; the link to the N-glycan core structure entry is assigned via the wwPDB entry.

Prior to the 2018 update, only glycans had been considered as entries in *Glycosciences.DB*. All other items such as literature references or wwPDB structures were only displayed as parts of the glycan entries or in search result lists. Now wwPDB structures and publications also receive individual entry pages, which display more data than in the former release (Figure 1). The three types of entries, i.e. glycans, publications and wwPDB structures, are cross-linked with each other. For each entry type an individual symbol is used, that is displayed in the header of the entry and also used in cross-links and search results lists, so that users can directly see what kind of entry will be opened in a link.

New wwPDB entries are added weekly by downloading newly released structures from the wwPDB and searching them for carbohydrate moieties. This process is mostly automatic. Human intervention is only required in case of potential problems, such as mismatches between the wwPDB residue name and the residue that is actually present in the 3D structure (14), or newly introduced wwPDB residue names for which no definition is stored in pdb2linucs (15) and pdb-care (16), the tools used to detect and validate the glycans in wwPDB structures. The primary citation of a wwPDB entry is also imported from the wwPDB and stored in *Glycosciences.DB*. This way wwPDB entries can be automatically linked with both glycan and literature entries. Cross-links between the latter two types of entries cannot be added automatically in a dependable manner, as there is no tool available that can reliably extract information on relevant carbohydrates from a publication. Nevertheless, the primary reference of a wwPDB entry often also deals with the carbohydrates in that entry, in particular in case of protein–carbohydrate complexes, where the carbohydrate moieties have been added on purpose and thus are usually (but not certainly) also an important topic of the publication. This is not necessarily the case with glycoproteins, where the glycans also might be a major topic of the publication, but often (particularly in case of short, truncated glycans) are just stated as “also detected” or even not mentioned at all. Therefore, cross-links between glycan and literature entries that are assigned via wwPDB entries are not listed together with manually assigned cross-links, but in a separate section, so that users can identify them easily.

### Glycosciences.DB glycan structure entries

Glycan structure entries (Figure 1 front) still form the main part of *Glycosciences.DB* content. The entries collect information on a carbohydrate structure, such as 3D structure models, NMR spectra, literature references, references to wwPDB entries and information on residue composition, substructure motifs, trivial names and taxonomy data. The 2018 update comes along with some further items. The glycan structure information (monosaccharid sequence and linkage positions) was only given in a 2D annotation in CarbBank format so far. Now we also offer the structure in LInear Notation for Unique description of Carbohydrate Sequences (LINUCS) notation (17), the notation internally used in the database to store and identify the glycan structures, and, where possible, in GlycoCT_condensed and GlycoCT_xml format (18). For further information on glycan structure formats, please refer to (19–21). In addition to these text formats, Symbol Nomenclature For Glycans (SNFG) graphs (22) have also been added to many glycan entries. At the time of writing, however, not all of the newly defined features of the current SNFG version are incorporated yet.

Cross-links to corresponding entries of other databases of the *Glycosciences.de* portal (*GlycoMapsDB* (23) and *GlycoCD* (24)) are also given now where applicable. A feature that is used by many genomics, proteomics or literature databases but to our knowledge not yet by glycomics databases is the option to add keywords to a database entry, which can be used to identify that entry in a database search. This option is implemented now in *Glycosciences.DB*. In analogy to literature entries and wwPDB entries, titles can now be added to glycan structure entries in *Glycosciences.DB*. It will be hardly possible to add meaningful titles to all entries. Nevertheless, there are various glycans for which trivial names are widely used (e.g. for Lewis-type blood group antigens (25), human milk oligosaccharides (26), glycosphingolipids of the ganglio series (27), etc.), and for many other glycans a brief description such as ‘core-fucosylated N-glycan core structure’ can be helpful for users who are not yet familiar with glycan structures. These titles can be used in database queries as well, and they are displayed together with the glycan structure in structure query results and in structure lists e.g. in literature entries to help users to identify the displayed glycans.

### Residue highlighting in 3D structure models

**Figure 2.**
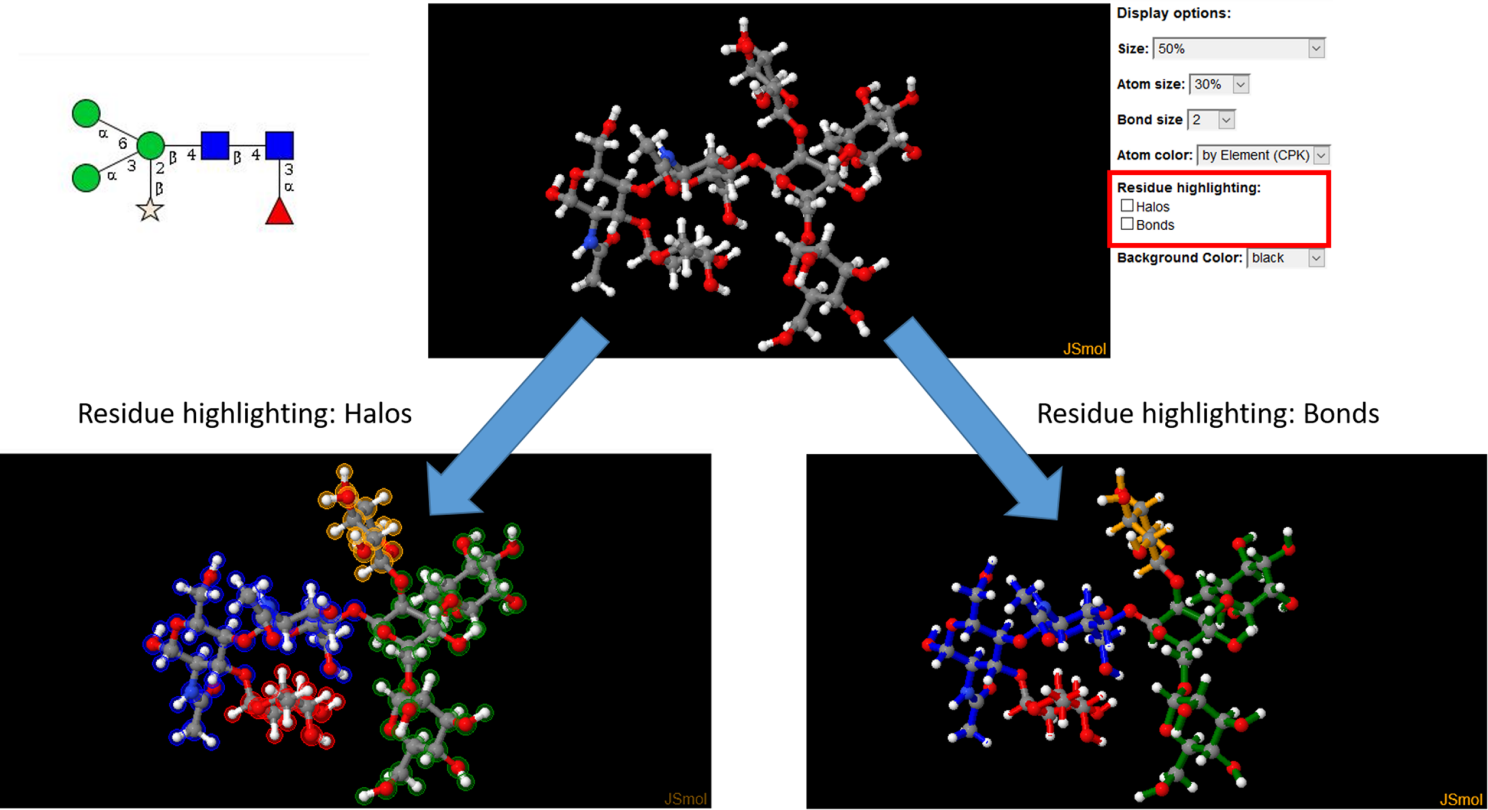
Residue highlighting in a plant N-glycan with core fucosylation and xylose (LinucsID 13934). Without highlighting, the residues are difficult to identify (top). This becomes easier when halos (bottom left) or bond colors (bottom right) are used with colors matching those of the SNFG symbols, even when the structure is oriented in a different way than the SNFG symbols.

The 3D structure models that are provided with many entries can give researchers a perception of what the glycans look like. However, it can be difficult to read a glycan’s 3D structure and find a specific residue within the structure, because the monosaccharide building blocks that form the glycans are very similar to each other. Therefore, we have added an option to color-highlight the residues using the colors of the SNFG symbols, which makes it easier to orient oneself in a glycan 3D structure (Figure 2). Halos or bond colors can be toggled with the check-boxes in the display options next to the 3D structure. So far, colors are set by PDB 3-letter codes for frequently occurring residues. The list of supported 3-letter codes will be further extended to cover more residues in the future.

### Content summaries in glycan structure lists

**Figure 3.**
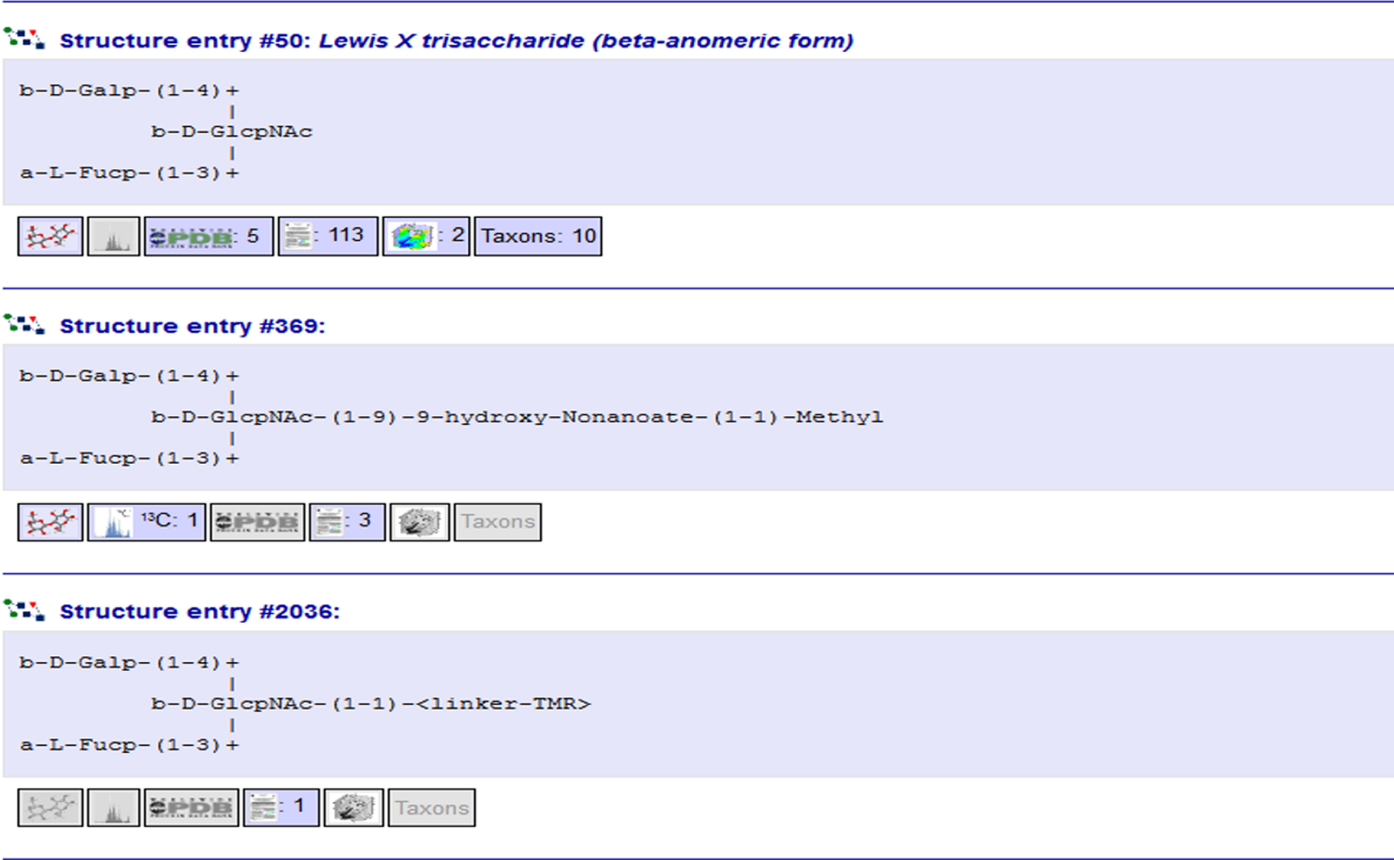
The content summary that is provided with each entry in glycan structure lists offers an overview of the data that are available for that entry. The entry on the top contains a 3D structure model, references to 5 wwPDB entries, 113 literature entries and 2 GlycoMaps, and 10 taxonomy items. The entry in the middle contains a 3D structure model, one 13C NMR spectrum and 3 literature references, and the bottom entry contains 1 literature reference but no further data.

The content of the individual glycan structure entries varies both in the kind of data that are available and in the extensiveness of the data. Therefore, a content summary has been added to each glycan in lists of glycan structure entries, such as search results, links to glycan entries from literature or wwPDB entries, or lists of corresponding other glycans with identical carbohydrate moieties in glycan entry pages. In this summary the user can see whether a 3D structure model is available for that entry, and how many NMR spectra, wwPDB entries, literature entries, glycomaps and taxonomy data are present (Figure 3). This helps users to identify entries with the information they are interested in without having to open and examine each entry manually.

### New search options in Glycosciences.DB

**Figure 4.**
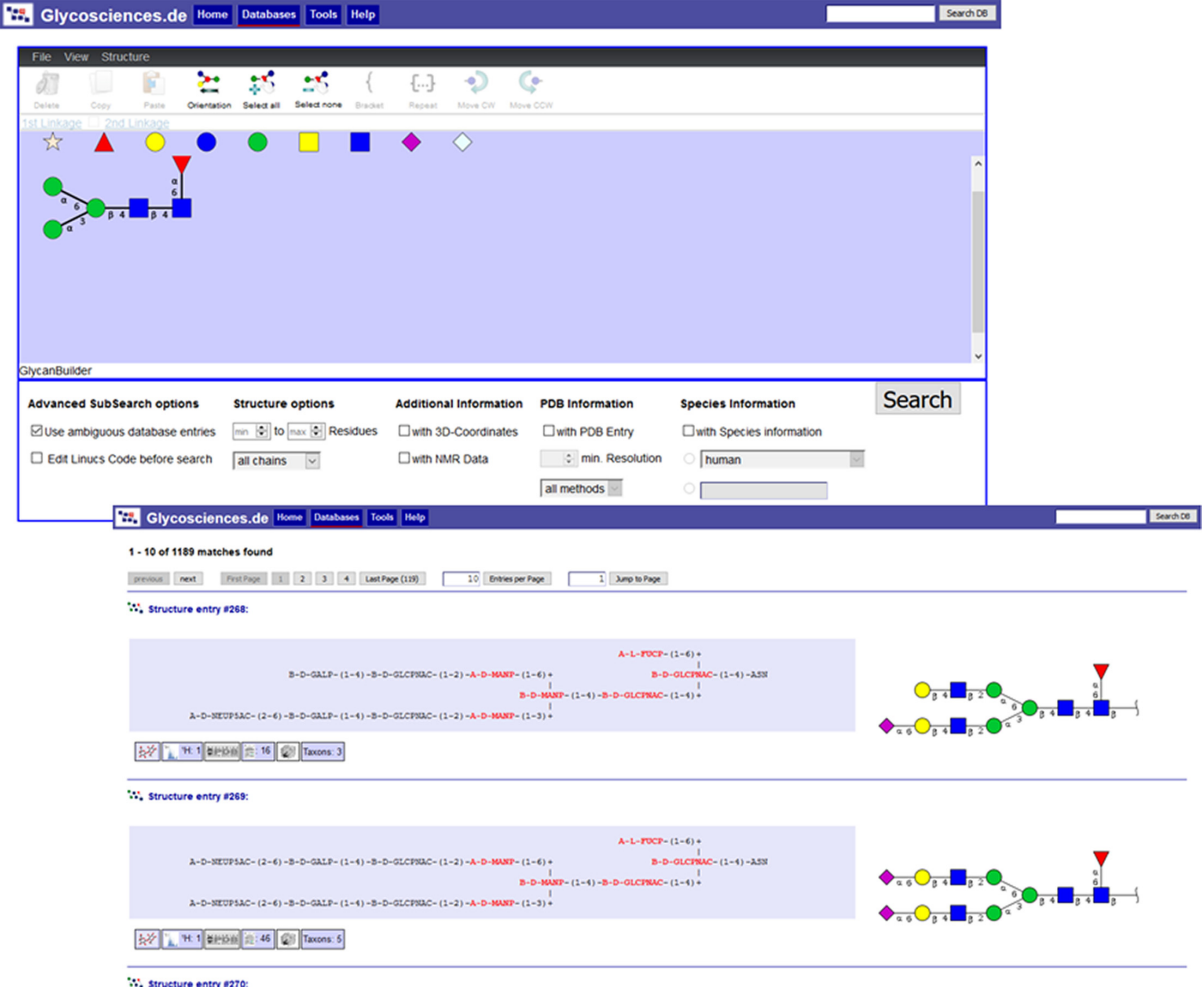
*GlycanBuilder* is used to create (sub-)structure search queries graphically (top). The results contain textual and SNFG representations of glycans next to each other (bottom). Whereas the symbols are easy to read for many glycoscientists, the text can be read without knowledge of the symbols. Furthermore, the text offers a highlighting of the substructure that matches the query, and it gives information on aglycones, which are not included in the symbols.

In comparison with the previously published options to find specific entries in *Glycosciences.DB* (8,28), two major improvements have been incorporated. First, the substructure search has been redesigned. In addition to the text-based input matrix, which is limited to five residues, *GlycanBuilder* (29) has been added as a graphical interface to create structure queries using SNFG-like symbols (Figure 4). A text field to type in or copy/paste structures in *CarbBank* or LINUCS notation is also available as an input for the substructure search. Second, a keyword based search that locates glycan structures, literature and wwPDB references in a single query is available now. This query can be accessed from any *Glycosciences.DB* page by typing the query text into the text input field in the header area of the page and clicking the “Search DB” button. It can also be used to quickly access a specific entry by using its ID as a search term.

### Substructure search algorithm

To conduct glycan substructure queries beyond the former limitation of five residues, a new search algorithm has been designed. Classical bioinformatics algorithms that have been designed for nucleotide or protein sequences cannot be applied to carbohydrate structures because of the various linkages between glycan residues and their ability to form branched structures. Instead, a graph comparison needs to be executed. Our algorithm works in a two-step process. In the first step, a set of potential matches is filtered based on the composition of the query structure. For example, if a query for a Lewis X trisaccharide consisting of one a-L-Fucp, one b-D-Galp and one b-D-GlcpNAc residue is executed, only structures containing at least one of each of these three residues need to be further analyzed. In the second step, the graph matching is performed treating the identified candidate structures. A notable feature of our implementation of the graph matching is that it takes into account repeating units. For example, a query consisting of six (2-8)-linked a-D-Neup5Ac residues also matches a glycan structure entry that is defined as [8)-a-D-Neup5Ac-(2-8)-a-D-Neup5Ac-(2-]_n_. This is also important for repeating units of heteropolysaccharides such as glycosaminoglycan structures. For example, a repeating unit that is defined as [4)-b-D-GlcpN-(1-4)-a-L-IdopA-(1-]_n_ is found by a query for b-D-GlcpN-(1-4)-a-L-IdopA as well as for a-L-IdopA-(1-4)-b-D-GlcpN, whereas a standard graph matching would only find the entry if the same residue order as in the database entry is used in the query. This means that with our algorithm the user does not need to know in which order the residues of the repeating unit are stored in the database.

Carbohydrate structures with a free reducing end have no fixed ring closure in solution, but due to mutarotation the reducing end residue switches between open chain, pyranose and furanose ring state and between α and β anomers when forming the ring. Therefore, many *Glycosciences.DB* glycan structure entries that are not linked to an aglycone are listed without anomer or ring definition, such as D-GlcNAc or D-Man. *GlycanBuilder*, however, uses pyranose rings as default ring type setting for most residues. That means that the query terms generated by *GlycanBuilder* contain the ring type symbol, as in D-GlcpNAc or D-Manp, and would not match the entries where no ring is defined. In some entries, anomer or ring type is not defined for non-reducing end residues as well. Nevertheless, all these entries can provide valuable information to a user. To find such entries as well without having to perform multiple queries, the search interface provides an option “Use ambiguous database entries”. If this option is active, then the algorithm matches a structure even if the query residue includes an anomer or ring type but the database entry residue does not.

### NMR spectra search

NMR spectra stored in *Glycosciences.DB* can be queried by residue and atom names or by chemical shifts (peak search). These queries have been described in detail before (30). The peak search in *Glycosciences.DB* can be performed for either ^1^H or ^13^C peaks. Recently, Klukowski and Schubert developed an algorithm that offers a peak search by both ^1^H and ^13^C peaks using NMR spectra from *Glycosciences.DB* (31). Although this search option is an external service, it is linked from *Glycosciences.DB*, and the search results link back to *Glycosciences.DB* structure entries.

### User-driven input

There are many possible ways to add new data to a database. Mostly these procedures are fully automated imports, supervised imports or manual additions. Imports of glycans from wwPDB entries are partly automated and, when issues are detected by the automatic process, supervised. Automatic or supervised imports are not yet reliably possible for other types of information, such as new literature references, titles or keywords to glycan structure entries. High-quality data of this kind can only be obtained by manual additions, which can be done only to a limited extent by a small team of researchers and developers. Therefore, *Glycosciences.DB* provides an easy-to-use web form where database users can add information to a glycan structure entry. As a first step, structure title, keywords and publications can be added. For the latter, only a PubMed ID is required, via which publication data can be extracted from *PubMed* (https://www.pubmed.gov/). Data can be added via the menu in the upper right corner of a glycan entry page. To avoid spamming, the entered information is not directly displayed in the entry but reviewed by the developers of *Glycosciences.DB* before it is published. There is also an option to leave a comment. This way users can indicate wrong annotation or add information, which is not yet covered by an individual interface.

## CONCLUSION AND OUTLOOK

The newly introduced features enable users to easily find information that is relevant to them. Furthermore, the entry pages for glycan-containing wwPDB entries in *Glycosciences.DB* provide an option to establish cross-links between proteomics and glycomics resources. Links from glycomics to proteomics databases are already present, but the opposite direction is rarely linked (20). *Glycosciences.DB* provides a list of the wwPDB IDs for which glycan data are available, so that any resource that stores wwPDB data can establish links to the corresponding entries in *Glycosciences.DB*. To improve cross-linking options between *Glycosciences.DB* and other glycomics resources we will register the entries that could be translated to GlycoCT_condensed notation in the *GlyTouCan* (32) repository, so that they are accessible via GlyTouCan IDs as well.

Various carbohydrate database projects such as CCSD/*CarbBank* or *EUROCarbDB* (33) have been discontinued after funding had stopped. Although *Glycosciences.DB* is no longer backed by a university position or a grant for ∼2 years now, it is still online and even further growing. A major reason for this is the mostly automatic update with data extracted from newly released wwPDB structures. Nevertheless, as mentioned above, even basic curation tasks require manual input from experts. Therefore, we encourage users of the database to pay back to the community by adding information to entries of their area of expertise. If this works well, we will extend the options for users of the database to provide further types of information.

*Conflict of interest statement*. None declared.
